# Factor XI(a) Inhibition in Secondary Stroke Prevention: On the Cusp of a New Anticoagulation Paradigm

**DOI:** 10.1021/acsptsci.6c00298

**Published:** 2026-09-11

**Authors:** Rami A. Al-Horani

**Affiliations:** Division of Basic and Pharmaceutical Sciences, College of Pharmacy, Xavier University of Louisiana, New Orleans, Louisiana 70125, United States

**Keywords:** Ischemic stroke, milvexian, abelacimab, osocimab, asundexian, fesomersen

## Abstract

Ischemic stroke remains a leading contributor to global mortality and long-term disability, while bleeding complications, particularly intracranial hemorrhage, continue to constrain the use of conventional antithrombotic therapies for secondary prevention. Factor XI (FXI) has emerged as an attractive therapeutic target because it promotes thrombin amplification and pathological thrombus stabilization while contributing only modestly to physiological hemostasis, forming the basis for “hemostasis-sparing” anticoagulation. Current FXI-targeted approaches include oral small-molecule FXIa inhibitors (asundexian and milvexian), antisense oligonucleotides (fesomersen), and monoclonal antibodies (abelacimab and osocimab). Among these, oral FXIa inhibitors appear particularly suited for long-term cerebrovascular prevention because they provide rapid, reversible target inhibition. Phase II trials, including PACIFIC-Stroke and AXIOMATIC-SSP, showed neutral primary efficacy end points but consistently favorable safety outcomes. More recently, the phase III OCEANIC-STROKE trial demonstrated that asundexian 50 mg once daily added to antiplatelet therapy reduced recurrent ischemic stroke by 26% (HR 0.74; 95% CI 0.65–0.84) without a significant increase in major bleeding among patients with noncardioembolic stroke. In contrast, OCEANIC-AF showed inferior efficacy compared with apixaban in atrial fibrillation, suggesting that the therapeutic value of FXIa inhibition is strongly dependent on clinical context. Taken together, this review summarizes the pathophysiological rationale, pharmacology, and clinical evidence supporting FXI inhibition for secondary ischemic stroke prevention and discusses future precision-medicine strategies for patient selection.

## Graphical Abstract

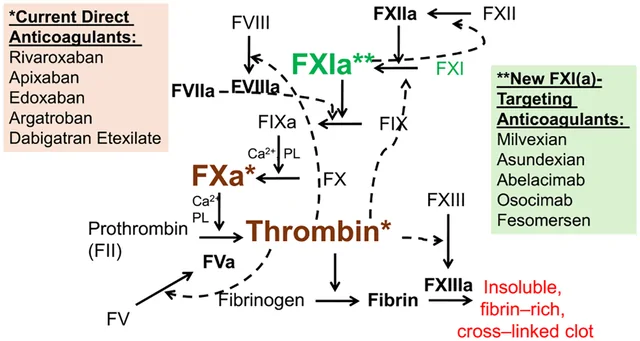

Ischemic stroke remains a major cause of global mortality and long-term disabilities. In 2021, stroke was the third most common cause of death globally, accounting for 7.3 million deaths (10.7% of all deaths) and 160.5 million disability-adjusted life years (DALYs), with 93.8 million prevalent and 11.9 million incident stroke events worldwide, according to the Global Burden of Disease 2021 study.^1^ Ischemic stroke constituted 65.3% of all incident strokes, with intracerebral hemorrhage (28.8%) and subarachnoid hemorrhage (5.8%) accounting for the remainder.^1^ The stroke incidence is projected to rise further, particularly in low- and middle-income countries, which account for more than 85% of stroke-related deaths and DALYs.^1^

Despite advances in secondary prevention, recurrent stroke remains common, with recurrence rates approaching 8% during the first year and 20–25% within 5 years, especially in patients with large-artery atherosclerosis or cardioembolic disease.^2,3^ Recurrent stroke is associated with increased mortality, disability, cognitive decline, and vascular dementia.^4^ Current antithrombotic strategies are effective but remain limited by bleeding risk, particularly intracranial hemorrhage (ICH), especially in elderly patients and those with cerebral small-vessel disease or microbleeds.^5,6^ In addition, 25–30% of ischemic strokes remain cryptogenic, and empirical anticoagulation has not shown superiority over antiplatelet therapy in embolic stroke of undetermined source (ESUS).^7,8^

Within this context, factor XI(a) (FXI(a)) inhibition has emerged as a promising therapeutic strategy. FXI amplifies thrombin generation and stabilizes pathological thrombi while playing a comparatively limited role in physiological hemostasis, supporting the concept of “hemostasis-sparing” anticoagulation. This review summarizes the biological rationale, pharmacology, and clinical evidence supporting FXI inhibition in secondary ischemic stroke prevention. It discusses future precision-based approaches for optimizing patient selection and benefit–risk balance. This narrative review was informed by a search of PubMed and Scopus for English-language articles published from 2000 to 2026 using terms including “factor XI,” “FXI inhibitor,” “asundexian,” “milvexian,” “abelacimab,” “osocimab,” “fesomersen,” and “secondary stroke prevention.” Reference lists of relevant articles were manually screened. Priority was given to randomized controlled trials, phase II/III clinical trials, and meta-analyses, with preclinical and mechanistic studies included for biological context.

## THE COAGULATION CASCADE AND CURRENT ANTITHROMBOTIC THERAPIES

The coagulation cascade is central to both physiological hemostasis and pathological thrombosis. In the classical model, coagulation is initiated through the extrinsic pathway, triggered by tissue factor (TF), and the intrinsic pathway, initiated by factor XII (FXII). Both pathways converge on factor X (FX), leading to thrombin generation, fibrin formation, and clot stabilization^9^ (Figure 1). Current anticoagulants target key components of this cascade. Vitamin K antagonists (VKAs) interfere with the vitamin K-dependent *γ*-carboxylation of glutamic acid residues on coagulation factors II, VII, IX, and X, thereby impairing their biological activity, whereas heparins enhance antithrombin-mediated inhibition of thrombin and FXa. Direct thrombin inhibitors such as dabigatran etexilate and argatroban block fibrin formation, while direct oral FXa inhibitors, including apixaban, edoxaban, and rivaroxaban, suppress FXa-mediated thrombin generation. Fondaparinux indirectly inhibits FXa through antithrombin activation.^10^ Direct oral anticoagulants (DOACs) improved anticoagulation management through more predictable pharmacokinetics, fewer interactions, and lower intracranial bleeding rates than VKAs. They are now first-line therapy for stroke prevention in nonvalvular atrial fibrillation.^11^ In a meta-analysis of 71,683 patients from the RE-LY, ROCKET-AF, ARISTOTLE, and ENGAGE AF-TIMI 48 trials, DOACs reduced stroke or systemic embolism by 19%, hemorrhagic stroke by 51%, and all-cause mortality by 10% compared with warfarin.^12^

In contrast to atrial fibrillation, anticoagulation is not routinely recommended for secondary prevention after non-cardioembolic ischemic stroke, where antiplatelet therapy remains the standard of care. This distinction reflects the unfavorable balance between ischemic stroke reduction and bleeding risk, particularly intracranial hemorrhage, when anticoagulants are added to antiplatelet regimens in patients without cardioembolic sources.^13^ The COMPASS trial evaluated very low-dose rivaroxaban (2.5 mg twice daily) plus aspirin in patients with stable atherosclerotic vascular disease, including those with prior stroke. While this combination reduced major adverse cardiovascular events compared with aspirin alone, it was also associated with increased major bleeding, including intracranial hemorrhage, and the net clinical benefit in stroke patients was uncertain.^14^ These findings highlight the ongoing need for antithrombotic strategies that provide additional protection against recurrent ischemic events without proportionally increasing bleeding risk, a gap that FXI(a) inhibition may potentially address.

Despite advances, bleeding remains a major limitation. Although DOACs reduce intracranial hemorrhage compared to VKAs, clinically relevant and major bleeding still occur. Among available DOACs, apixaban has the most favorable gastrointestinal safety profile and is the only agent not consistently associated with increased gastrointestinal bleeding versus warfarin.^15,16^ Evidence also remains limited in patients with severe renal impairment or dialysis because these populations were largely excluded from pivotal trials.^15^ In addition, DOACs are not routinely recommended during pregnancy or breastfeeding because of insufficient safety data.^17^ Therefore, safer anticoagulants that preserve antithrombotic efficacy while minimizing bleeding risk remain an important unmet need in secondary stroke prevention.

Importantly, the classical coagulation model (Figure 1A) has shifted toward a cell-based framework (Figure 1B) over the past two decades,^18^ in which coagulation occurs on cellular surfaces, primarily TF-bearing cells and activated platelets, and progresses through three overlapping phases of initiation, amplification, and propagation. In the initiation phase, TF exposure forms the TF–FVIIa complex, activating FX and generating limited thrombin sufficient to trigger fibrin formation and platelet activation. During amplification, thrombin activates factors V, VIII, and FXI on platelet surfaces, priming the system for robust clotting. In the propagation phase, activated platelets drive sustained thrombin generation via intrinsic pathway amplification, resulting in stable fibrin-rich thrombus formation.^19^ While physiological hemostasis and pathological thrombosis share the same core machinery, their phase dependency differs. Hemostasis is primarily driven by TF-mediated initiation with limited FXI involvement, whereas thrombosis depends more heavily on FXI/FXIa-driven feedback loops that sustain thrombin generation and thrombus growth.^20,21^ This mechanistic separation underlies the concept of “hemostasis-sparing” anticoagulation. Conventional agents targeting thrombin or FXa impair both thrombosis and hemostasis, whereas selective FXI(a) inhibition may preferentially reduce pathological thrombus propagation while preserving physiological clotting. This distinction is particularly relevant in cerebrovascular disease, where effective prevention of thromboembolism must be balanced against the risk of intracranial hemorrhage.

## EVOLUTION OF FXI(A) AS A DRUG TARGET

FXI is a 160-kDa disulfide-linked homodimeric serine protease zymogen. It is composed of two identical subunits, each containing four apple domains and a C-terminal catalytic domain mediating interactions with high-molecular-weight kininogen, thrombin, FIX, FXII, platelets, and heparin. Upon activation to FXIa, it cleaves FIX and amplifies thrombin generation via the intrinsic pathway^22^ (see ref Figure 1). FXI is activated via two main mechanisms. FXIIa-driven activation within the contact pathway and thrombin-mediated activation create a positive feedback loop sustaining thrombin generation.^20,21^ This loop is reinforced by activated platelets and polyanionic mediators, including extracellular nucleic acids, neutrophil extracellular traps, and platelet polyphosphates, in inflammatory and thrombotic environments.^20,23^

In physiological hemostasis, FXI plays a limited role because TF–FVIIa–driven initiation is sufficient to generate adequate thrombin for clot formation even in its absence.^21^ This is reflected clinically in congenital FXI deficiency (hemophilia C), which produces a mild, variable bleeding phenotype with rare spontaneous hemorrhage and bleeding mainly after surgery or trauma, particularly in high-fibrinolytic tissues such as the oral cavity, nasopharynx, and genitourinary tract.^24,25^ Therefore, FXI deficiency supports the concept that reduced FXI activity is associated with lower risk of venous thromboembolism^26^ and ischemic stroke,^27^ while causing only modest bleeding. In contrast, elevated FXI levels are linked to increased thrombotic risk, including more than a 2-fold increase in venous thromboembolism,^28^ and a causal association with ischemic stroke that has been confirmed by Mendelian randomization analyses (OR 2.54; 95% CI 1.68–3.84; p = 0.00001), particularly cardioembolic stroke.^29^ Higher FXI activity is also associated with recurrent vascular events and mortality after stroke.^30^

Beyond thrombin generation, FXIa enhances thrombus stability by activating thrombin-activatable fibrinolysis inhibitor (TAFI), generating fibrin clots that are more resistant to lysis.^31,32^ FXI inhibition increases endogenous fibrinolysis approximately 2-fold via reduced TAFI activation,^33^ suggesting a mechanistic basis for enhanced clot susceptibility to lysis. Additional data suggest roles for FXI in endothelial dysfunction and vascular inflammation independent of coagulation activity.^34–36^ Overall, converging structural, genetic, epidemiological, and experimental evidence identify FXI as a key driver of pathological thrombus amplification while being relatively dispensable for normal hemostasis, supporting its therapeutic targeting.

## FACTOR XI(A) INHIBITORS

FXI-targeted anticoagulants can be grouped into three main classes: oral small-molecule FXIa inhibitors, monoclonal antibodies, and antisense oligonucleotides (ASOs). While all reduce intrinsic-pathway thrombin amplification, they differ markedly in mechanism of action, pharmacokinetics, pharmacodynamics, route of administration, reversibility, and drug–drug interaction potential.^37^ Small molecules inhibit circulating FXIa directly and reversibly; monoclonal antibodies neutralize FXI and/or FXIa or block FXI activation; ASOs suppress hepatic FXI synthesis at the mRNA level. These mechanistic differences are clinically relevant, as they determine onset/offset kinetics, periprocedural management, bleeding reversal strategies, and suitability for chronic stroke prevention.^38^

## SMALL-MOLECULE ORAL FXIA INHIBITORS

Oral agents such as asundexian and milvexian currently represent the most clinically advanced FXI-directed therapies for long-term secondary stroke prevention due to their oral bioavailability and rapid, reversible FXIa inhibition (Table 1). Both are low-molecular-weight compounds (Figure 2) that selectively bind the FXIa catalytic site, inhibiting intrinsic-pathway thrombin amplification without reducing circulating FXI levels. Compared with ASOs and monoclonal antibodies, their faster onset and offset improve flexibility in elderly patients requiring frequent medication changes or temporary interruption for procedures.^39^ Their development required careful optimization of potency, selectivity, oral exposure, permeability, and metabolic stability, particularly given the structural constraints of the FXIa active site.^40,41^ Preclinical thrombosis models in rabbits and nonhuman primates consistently demonstrated that FXIa inhibition reduces thrombus formation with minimal effects on bleeding time at effective doses.^42–44^ Earlier compounds (e.g., BMS-262084, BMS-654457) and later agents (e.g., BMS-724296) showed dose-dependent antithrombotic efficacy, modest APTT prolongation, minimal PT changes, and lower bleeding liability compared with FXa or thrombin inhibitors.^42–44^ These findings supported APTT as a pharmacodynamic marker of FXIa inhibition and suggested a potentially wider therapeutic window than conventional common-pathway anticoagulants.^21,59^

Both asundexian and milvexian produce concentration-dependent FXIa inhibition with parallel prolongation of APTT, while PT remains largely unchanged, confirming selectivity for the intrinsic pathway. For asundexian, FXIa inhibition is rapid and exposure-dependent. In PACIFIC-AF, 20 mg reduced FXI activity by ~81–90%, while 50 mg achieved ~92–94% inhibition across trough and peak levels.^61^ Milvexian similarly produces dose-dependent reductions in FXI activity and APTT prolongation.^50,62^ Across both programs, APTT has served as the principal pharmacodynamic marker of target engagement. However, no validated exposure–response or APTT threshold has yet been established to predict clinical efficacy or bleeding in secondary stroke prevention.

On one hand, asundexian exhibits high oral bioavailability (~94%).^45,46^ Phase I studies demonstrated approximately dose-proportional exposure across 25–100 mg once-daily dosing, with low-to-moderate interindividual variability and minimal accumulation after repeated administration.^46,47^ Single-dose administration (5–150 mg) produced dose-dependent exposure with a terminal half-life of ~14–18 h, supporting once-daily dosing.^39,46^ Median *t*_max_ is ~1–1.5 h for oral solution and 2–4 h for immediate-release tablets. Food, gastric pH modification, and formulation have minimal impact on overall exposure, although high-fat meals and antacids may modestly delay absorption.^46^ Distribution is mainly intravascular, with an apparent steady-state volume of distribution of ~70.5 L.^46^ Asundexian is a low-clearance compound with negligible first-pass metabolism.^63^ Mass-balance studies show near-complete recovery of radioactivity, primarily via feces (~80%) and to a lesser extent urine (~20%, including ~6% unchanged drug). Elimination is driven mainly by amide hydrolysis and excretion of the parent drug, with minimal oxidative metabolism.^63^ The major circulating metabolite (M10) is pharmacologically inactive.^64^

Metabolism is primarily mediated by carboxylesterase 1 (CES1), with minor CYP3A4 contribution, and the drug is also a P-glycoprotein substrate.^46^ Population pharmacokinetic analyses confirmed dose-proportionality and identified covariates (age, sex, body weight, renal function, and CYP3A4 inhibitors) as statistically significant but not clinically relevant.^47^ Drug–drug interaction studies showed that strong CYP3A4/P-gp inhibitor (itraconazole) increased exposure by ~103%, moderate CYP3A4/P-gp inhibitor (verapamil) increased exposure by ~76%, and moderate CYP3A4 inhibitor (fluconazole) had minimal effect.^63^ Carbamazepine (CYP3A4/P-gp induction) reduced exposure by ~44%.^63^ Overall, predominant CES1 metabolism may reduce clinically relevant interaction liability compared with many oral anticoagulants.

On the other hand, milvexian also demonstrates favorable oral pharmacokinetics, with key differences from asundexian. Phase I studies showed rapid absorption with *t*_max_ ~ 2.5–4 h under fasting conditions.^51^ Terminal half-life ranges from ~8.3–13.8 h after single dosing and ~11.4–18.1 h after multiple dosing,^51^ supporting once- or twice-daily regimens. Population studies in Japanese and Chinese cohorts showed comparable pharmacokinetics, with half-lives ~9–12 h.^50,52,62^ Steady-state is achieved within ~6 days of twice-daily dosing.^51^ Exposure is dose-proportional between 25–200 mg under fasting conditions, whereas high-dose administration with food delays absorption and increases exposure.^50,53^

Milvexian is primarily metabolized via CYP3A4/3A5 and is a P-gp substrate.^51^ Renal excretion accounts for <20% of clearance, with hepatobiliary elimination predominating. Compared with asundexian, it is more susceptible to drug–drug interactions. Itraconazole increases exposure by ~2.5-fold, while diltiazem produces smaller increases.^54^ Rifampin markedly reduces exposure via CYP3A/P-gp induction.^55^ These interactions are clinically relevant in stroke populations with frequent polypharmacy. Mild hepatic impairment has a limited effect on exposure,^56^ whereas moderate-to-severe renal impairment increases exposure by ~41–54% and prolongs half-life.^52^ No clinically meaningful ethnic differences in pharmacokinetics or pharmacodynamics have been observed.^50–52,62^ These differences underpin dosing strategies: asundexian supports once-daily administration due to longer half-life and stable exposure, whereas milvexian is generally optimized for twice-daily dosing to maintain more consistent 24-h FXIa inhibition.

## MONOCLONAL ANTIBODIES

Monoclonal antibodies targeting FXI and/or FXIa (Table 2), including abelacimab, osocimab, BAY 1831865, SHR-2004, REGN7508^Cat^, and REGN9933^A2^—provide rapid and sustained inhibition of the intrinsic coagulation pathway.^65–68^

Mechanistically, abelacimab binds the catalytic domain of FXI and stabilizes the inactive zymogen conformation, thereby preventing activation by both thrombin and FXIIa.^69^ Intravenous administration reduces free FXI levels by >99% within 1 h.^70^ In the AZALEA-TIMI 71 trial, monthly subcutaneous abelacimab produced sustained FXI suppression and substantially lower bleeding rates compared with rivaroxaban.^71^ Osocimab binds adjacent to the FXIa active site and prevents FIX activation.^73^ In the FOXTROT trial, among patients undergoing knee arthroplasty, postoperative osocimab 0.6 mg/kg, 1.2 mg/kg, and 1.8 mg/kg met criteria for noninferiority compared with enoxaparin, and the preoperative 1.8 mg/kg dose of osocimab met criteria for superiority compared with enoxaparin for the primary outcome of incidence of venous thromboembolism at 10 to 13 days postoperatively.^73^

These agents are administered intravenously or subcutaneously and exhibit prolonged half-lives ranging from approximately 12 to >40 days depending on the specific molecule.^39^ Unlike small-molecule inhibitors, monoclonal antibodies are eliminated primarily through proteolytic degradation within the reticuloendothelial system rather than hepatic or renal clearance pathways.^77^ Consequently, they exhibit minimal classical pharmacokinetic interaction liability and may be particularly useful in patients with advanced renal dysfunction. However, their long duration of action limits reversibility and may complicate the urgent surgery or management of major bleeding.^78^ Immunogenicity data remain limited. Abelacimab has not demonstrated clinically significant antidrug antibody formation in current studies,^67,68^ whereas osocimab produced antidrug antibodies of low titer in 65.0% of Chinese volunteers.^75^ Long-term surveillance remains important for chronically administered biologic therapies.

Similar to small-molecule FXIa inhibitors, monoclonal antibodies targeting FXI and/or FXIa prolong the APTT in a concentration-dependent manner, reflecting their inhibition of the intrinsic coagulation pathway.^63,64^ However, the magnitude and clinical utility of APTT prolongation differ among the agents. For abelacimab, the marked suppression of FXI activity (>99%) is associated with substantial APTT prolongation, although the relationship between APTT and antithrombotic efficacy or bleeding risk has not been fully established.^69,79^ Letunica et al. recently demonstrated that abelacimab produced dose-dependent APTT prolongation in vitro in pediatric plasma samples, with effects comparable to those observed in adults, suggesting consistent pharmacodynamic responses across age groups.^80^ In contrast, osocimab shows more modest APTT prolongation at therapeutic concentrations, reflecting its distinct binding epitope adjacent to the FXIa active site.^73,75^ The prothrombin time (PT) remains largely unaffected by FXI-directed monoclonal antibodies, consistent with their selectivity for the intrinsic pathway. A multicenter study evaluating FXI(a) inhibitors at clinically relevant concentrations demonstrated that APTT was prolonged in a concentration-dependent manner, with milvexian having the greatest impact, followed by abelacimab and asundexian.^81^ Importantly, no validated APTT threshold has been established to predict clinical efficacy or bleeding risk for any FXI-directed monoclonal antibody, and routine coagulation monitoring is not currently recommended in clinical practice.^39,78^

## ANTISENSE OLIGONUCLEOTIDES

Antisense oligonucleotides (ASOs) differ fundamentally from oral FXIa inhibitors because they suppress hepatic FXI synthesis through targeted degradation of FXI mRNA rather than direct inhibition of circulating FXIa.^19,57^ Major agents in this class include IONIS-FXIRx and fesomersen (Table 3).

These agents are administered subcutaneously and exhibit prolonged elimination half-lives, ranging from approximately 1–2 weeks for IONIS-FXIRx to 10–20 days for fesomersen.^65^ Fesomersen incorporates GalNAc conjugation, which enhances hepatocyte uptake through the asialoglycoprotein receptor and enables lower dosing with monthly administration.^74^ Both ASOs demonstrate high plasma protein binding (>90%) and minimal dependence on CYP450 metabolism. In the RE-THINC trial, fesomersen produced dose-dependent reductions in FXI levels in patients receiving hemodialysis.^82^ Because ASOs undergo nuclease-mediated degradation rather than CYP-mediated metabolism, clinically significant pharmacokinetic drug–drug interactions are expected to be minimal. The prolonged pharmacodynamic effects of ASOs may be advantageous for patients requiring stable long-term anticoagulation, although their delayed onset and offset reduce flexibility during urgent procedures or bleeding events.

Considering the above findings, it appears that pharmacokinetic differences among FXI inhibitors have the most clinical implications. A key distinction is the contrast between short-acting oral FXIa inhibitors and long-acting parenteral FXI-lowering agents. Oral agents such as asundexian and milvexian provide rapid onset, shorter half-lives, and easier temporary interruption, features particularly advantageous in elderly patients with ischemic stroke who frequently require procedures or medication adjustments.^39,78,83^ However, oral FXIa inhibitors remain susceptible to CYP3A4- and P-gp-mediated drug interactions, especially with antifungals, antiarrhythmics, and enzyme-inducing antiseizure medications. In contrast, ASOs and monoclonal antibodies provide sustained FXI suppression with infrequent dosing and minimal metabolic interaction liability but are considerably less flexible during urgent bleeding or invasive procedures.^78,84^ These pharmacokinetic differences also influence bleeding management. Short-acting oral agents may often be managed through temporary discontinuation alone, whereas reversal of long-acting therapies remains more challenging. Potential strategies proposed for bleeding associated with FXI inhibition include FXI concentrate, tranexamic acid, and low-dose recombinant FVIIa.^78^ Overall, although all FXI-targeted approaches support the concept of “hemostasis-sparing” anticoagulation, small-molecule oral FXIa inhibitors currently appear most suitable for long-term secondary prevention after ischemic stroke.

In addition to these three major classes, several other FXI-directed modalities are under investigation, although they are at earlier stages of clinical development. Nucleic acid aptamers, such as the RNA aptamer 11.16 and its derivatives, selectively bind FXIa and inhibit its activity through allosteric or direct mechanisms, offering the potential for rapid reversal via complementary antidote oligonucleotides.^85^ Natural inhibitors, including Kunitz-type serine protease inhibitors from hematophagous organisms (e.g., the tick-derived inhibitor Ir-CPI), provide structural insights into FXIa inhibition and may inform future drug design, though their therapeutic application remains limited by immunogenicity and pharmacokinetic challenges.^86^ Small interfering RNA (siRNA) approaches targeting hepatic FXI synthesis represent a conceptually similar strategy to ASOs, with the potential for sustained FXI knockdown following infrequent administration.^87^ However, siRNA therapeutics for FXI have not yet advanced to clinical trials. While these modalities are not yet poised for clinical use in stroke prevention, they underscore the diversity of approaches being explored to achieve “hemostasis-sparing” anticoagulation.

## CLINICAL TRIALS INVOLVING FXI(A) INHIBITION FOR SECONDARY PREVENTION AFTER ISCHEMIC STROKE, AS WELL AS META-ANALYSES

Clinical development of FXI-targeted therapies has increasingly shifted toward indication-specific applications, with the strongest evidence currently being derived from oral small-molecule FXIa inhibitors. FXI inhibition may provide benefit across several cerebrovascular settings, including secondary prevention after noncardioembolic ischemic stroke, atrial fibrillation, and thrombotic conditions associated with extracorporeal circuits or implanted devices.

PACIFIC-Stroke (NCT04304508) was an international, randomized, double-blind, placebo-controlled phase IIb trial that enrolled 1,808 patients aged ≥ 45 years with recent noncardioembolic ischemic stroke within 48 h of symptom onset.^83^ Participants received asundexian 10, 20, or 50 mg once daily or placebo in addition to standard antiplatelet therapy. The primary efficacy end point, a composite of recurrent symptomatic ischemic stroke and MRI-detected covert brain infarction, was neutral, with similar event rates across treatment groups and no significant dose–response relationship.^83^ Importantly, covert infarctions accounted for approximately 75% of outcome events, potentially obscuring clinically meaningful reductions in symptomatic stroke recurrence. Subsequent MRI analyses suggested that asundexian 50 mg may reduce recurrent ischemic events among patients with imaging features consistent with embolic or large-artery disease (HR 0.71; 95% CI 0.45–1.11), whereas no clear benefit was observed in isolated small subcortical infarcts.^88^ Despite neutral efficacy findings, safety outcomes were favorable. Major or clinically relevant nonmajor bleeding did not significantly increase compared with placebo (pooled HR 1.57; 90% CI 0.91–2.71).^89^ In addition, asundexian was not associated with increased cerebral microbleeds or hemorrhagic transformation.^90,91^

The subsequent phase III OCEANIC-STROKE trial (NCT05686070) was specifically designed to address limitations identified in PACIFIC-Stroke.^92^ This randomized, double-blind, event-driven study evaluated asundexian 50 mg once daily versus placebo on top of standard single or dual antiplatelet therapy in patients with recent noncardioembolic ischemic stroke or high-risk transient ischemic attack. Between January 2023 and February 2025, 12,327 patients were randomized. The trial met both primary efficacy end points, with asundexian reducing recurrent ischemic stroke compared with placebo (6.2% vs 8.4%; HR 0.74; 95% CI 0.65–0.84; *p* < 0.001), corresponding to a 26% relative risk reduction and an absolute risk reduction of approximately 1.9% at one year.^92^ Benefits were consistent across prespecified subgroups, including age, sex, stroke severity, etiologic subtype, and antiplatelet strategy. Asundexian also reduced composite cardiovascular outcomes as well as disabling or fatal stroke.^92^ Importantly, these efficacy benefits were achieved without a statistically significant increase in major bleeding (1.9% vs 1.7%; HR 1.10; 95% CI 0.85–1.44), symptomatic intracranial hemorrhage, hemorrhagic stroke, or fatal bleeding.^92^ These findings strongly support the concept of “hemostasis-sparing” anticoagulation in secondary stroke prevention.

In contrast, the OCEANIC-AF trial (NCT05643573) demonstrated that FXIa inhibition alone was inferior to apixaban for stroke prevention in atrial fibrillation.^93^ Asundexian 50 mg once daily was associated with substantially higher rates of stroke or systemic embolism compared with apixaban (1.3% vs 0.4%; HR 3.79; 95% CI 2.46–5.83), despite lower rates of major bleeding. These findings suggest that the efficacy of FXI inhibition is highly context dependent. In atrial fibrillation, where tissue factor–driven thrombin generation predominates and DOACs are highly effective, selective FXIa inhibition alone may provide insufficient anticoagulant intensity. Conversely, among patients with noncardioembolic stroke already receiving antiplatelet therapy, lower-intensity FXIa inhibition may offer clinically meaningful protection with lower bleeding risk.

Milvexian has been evaluated primarily in the AXIOMATIC-SSP phase II trial (NCT03766581), which enrolled 2,366 patients with recent ischemic stroke or high-risk transient ischemic attack (TIA).^94^ Participants received a placebo or various milvexian dosing regimens in addition to dual antiplatelet therapy. Similar to PACIFIC-Stroke, the primary composite end point of symptomatic stroke plus covert MRI infarction was neutral. However, symptomatic ischemic stroke rates were numerically lower in most milvexian treatment groups, particularly at intermediate doses.^94^ These findings again suggested that covert infarction end points may have diluted clinically relevant reductions in symptomatic stroke recurrence. Major bleeding rates remained low across treatment groups, although modest increases in nonmajor bleeding were observed at higher milvexian doses.^94^ Lastly, the ongoing phase III LIBREXIA-STROKE trial (NCT05702034) was designed on the basis of these findings and is evaluating milvexian 25 mg twice daily, the regimen considered to provide the most favorable balance between efficacy and bleeding risk. The results are anticipated by the end of 2026.

Several meta-analyses have evaluated the evolving evidence surrounding FXI/XIa inhibitors, although interpretation remains complicated by heterogeneity in patient populations, background antithrombotic therapy, and trial design. Early stroke-focused analyses demonstrated favorable safety profiles with uncertain efficacy signals. Palaiodimou et al.,^95^ Galli et al.,^96^ and Franco-Moreno et al.^97^ each reported trends toward reduced recurrent ischemic stroke without significant increases in major bleeding or intracranial hemorrhage. A larger meta-analysis by Zahid et al.,^98^ which included 14 randomized trials involving more than 30,000 patients, demonstrated a significant reduction in major bleeding with FXI/XIa inhibitors (RR 0.47; 95% CI 0.33–0.66), with a trend toward lower intracranial hemorrhage. However, efficacy varied substantially according to clinical setting. In atrial fibrillation populations, FXI/XIa inhibitors were associated with increased ischemic stroke risk compared with DOACs,^99,100^ findings largely driven by the negative OCEANIC-AF results. Together, current evidence suggests that FXI inhibition may provide the greatest clinical benefit in patients with residual thrombotic risk despite antiplatelet therapy, but in whom conventional anticoagulation is limited by bleeding risk.

The favorable bleeding profile observed across FXI inhibitor trials, together with the positive findings of OCEANIC-STROKE, highlights the importance of precision-based patient selection. Patients most likely to benefit may include those with persistent ischemic risk despite optimized antiplatelet therapy but with concomitant hemorrhagic vulnerability that precludes escalation to conventional anticoagulation. Cerebral microbleeds (CMBs) represent a particularly important imaging biomarker in this context. The MICON individual-patient-data meta-analysis demonstrated that patients with ≥5 CMBs after ischemic stroke or TIA have a substantially increased risk of symptomatic intracranial hemorrhage during antithrombotic therapy.^5^ Similarly, composite cerebral small-vessel disease scores incorporating lacunes, white-matter hyperintensities, enlarged perivascular spaces, and CMBs independently predict recurrent stroke and intracranial hemorrhage,^101,102^ while prior hemorrhagic transformation reflects increased neurovascular fragility.^103,104^

Conversely, patients with symptomatic large-artery atherosclerosis, embolic imaging patterns, multiple acute infarcts in different vascular territories, or branch atheromatous disease may derive greater benefit from additional anticoagulant mechanisms.^105^ These observations align with exploratory MRI analyses from PACIFIC-Stroke, which suggested greater benefit among patients with embolic or large-artery imaging features.^88^ Conventional risk scores such as CHA_2_DS_2_–VASc and HAS-BLED perform poorly for predicting intracranial bleeding because many predictors contribute simultaneously to ischemic and hemorrhagic risk.^106^ Biomarkers of vascular inflammation, including IL-6 and high-sensitivity C-reactive protein (hsCRP), may further refine patient stratification.^107^ Overall, current evidence supports a multimodal precision medicine approach integrating clinical variables, neuroimaging findings, and circulating biomarkers to identify patients most likely to achieve net clinical benefit from FXIa inhibition.

## CONCLUSIONS

FXI has emerged as one of the most promising anticoagulant targets for secondary stroke prevention because it plays a central role in thrombin amplification and pathological thrombus stabilization while contributing relatively little to physiological hemostasis.^108–111^ This biological dissociation forms the basis of the “hemostasis-sparing” anticoagulation paradigm. The phase III OCEANIC-STROKE trial provided the first evidence that oral FXIa inhibition can reduce recurrent ischemic stroke in patients with noncardioembolic stroke receiving antiplatelet therapy, demonstrating a 26% relative risk reduction without a significant increase in major or intracranial bleeding.^92^ In contrast, the negative findings of OCEANIC-AF showed that FXIa inhibition alone was inferior to apixaban for stroke prevention in atrial fibrillation,^93^ indicating that the efficacy of FXI inhibition is highly dependent on the underlying thrombotic mechanism and clinical context. Overall, current evidence suggests that FXIa inhibition may be particularly valuable in patients with noncardioembolic stroke who remain at residual ischemic risk despite antiplatelet therapy but are also vulnerable to bleeding complications with conventional anticoagulation. The ongoing LIBREXIA-STROKE trial will further clarify whether milvexian can confirm this therapeutic strategy.^94^

Future implementation of FXI-directed therapy will likely depend on precision-based approaches integrating clinical characteristics, neuroimaging findings, and inflammatory or hemostatic biomarkers to optimize patient selection and net clinical benefit.^5,101–107^ Rather than universally replacing existing antithrombotic therapies, FXI inhibitors may ultimately occupy an important niche in patients who are insufficiently protected by current strategies yet are unable to tolerate more intensive anticoagulation.

## Figures and Tables

**Figure 1.**
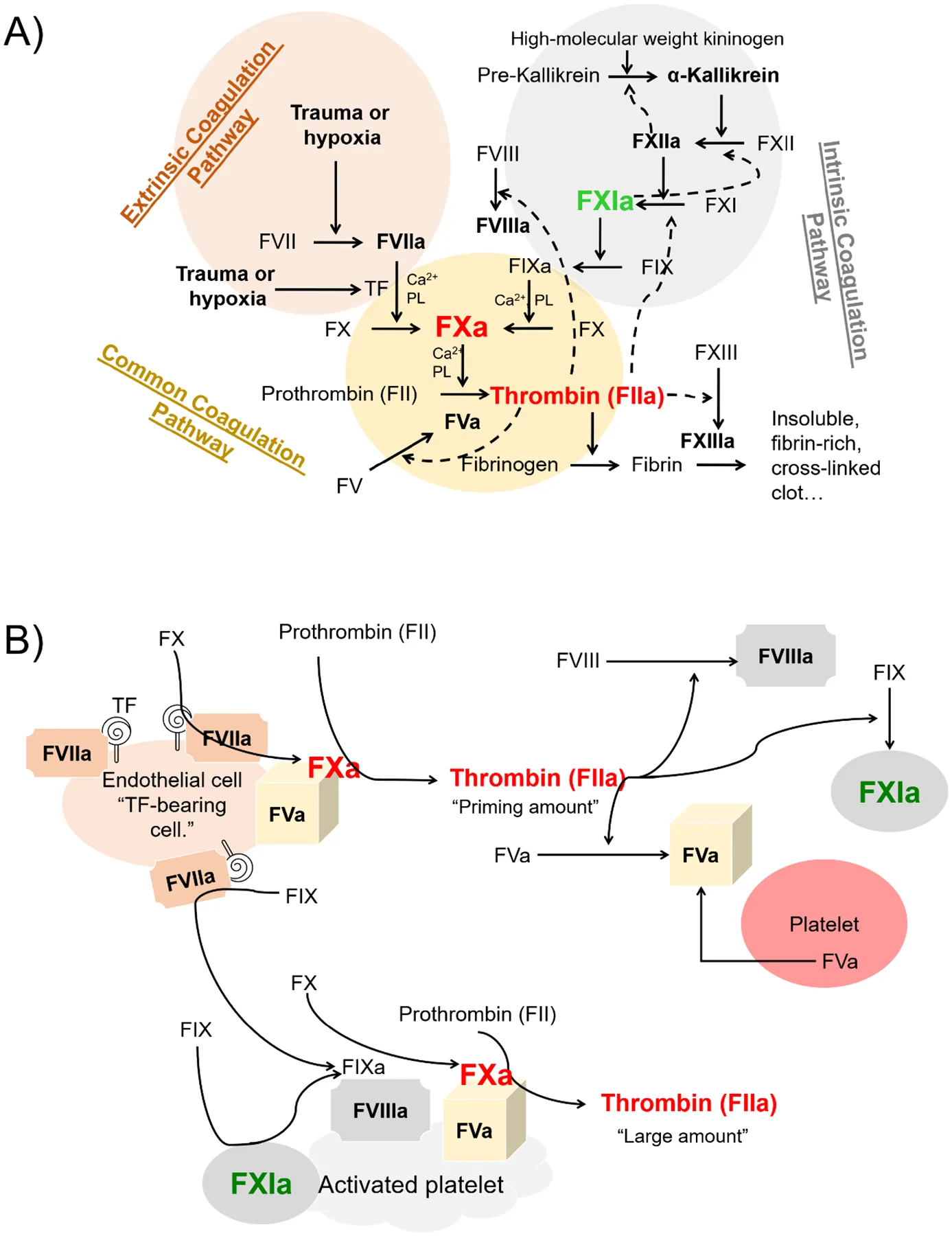
(A) Classical waterfall model of coagulation. The extrinsic pathway (FVIIa/TF), intrinsic pathway (FXIIa, FXIa, FIXa), and common pathway (FXa and thrombin) operate sequentially in a proteolytic cascade culminating in fibrin formation and FXIIIa-mediated clot stabilization. (B) Cell-based model of coagulation. Coagulation is initiated on TF-bearing cells, where the TF–FVIIa complex activates FX and generates small amounts of thrombin. During amplification, thrombin activates platelets, cofactors, and FXI through positive feedback mechanisms. The propagation phase occurs on activated platelet surfaces and produces the thrombin burst required for stable clot formation and effective hemostasis.

**Figure 2.**
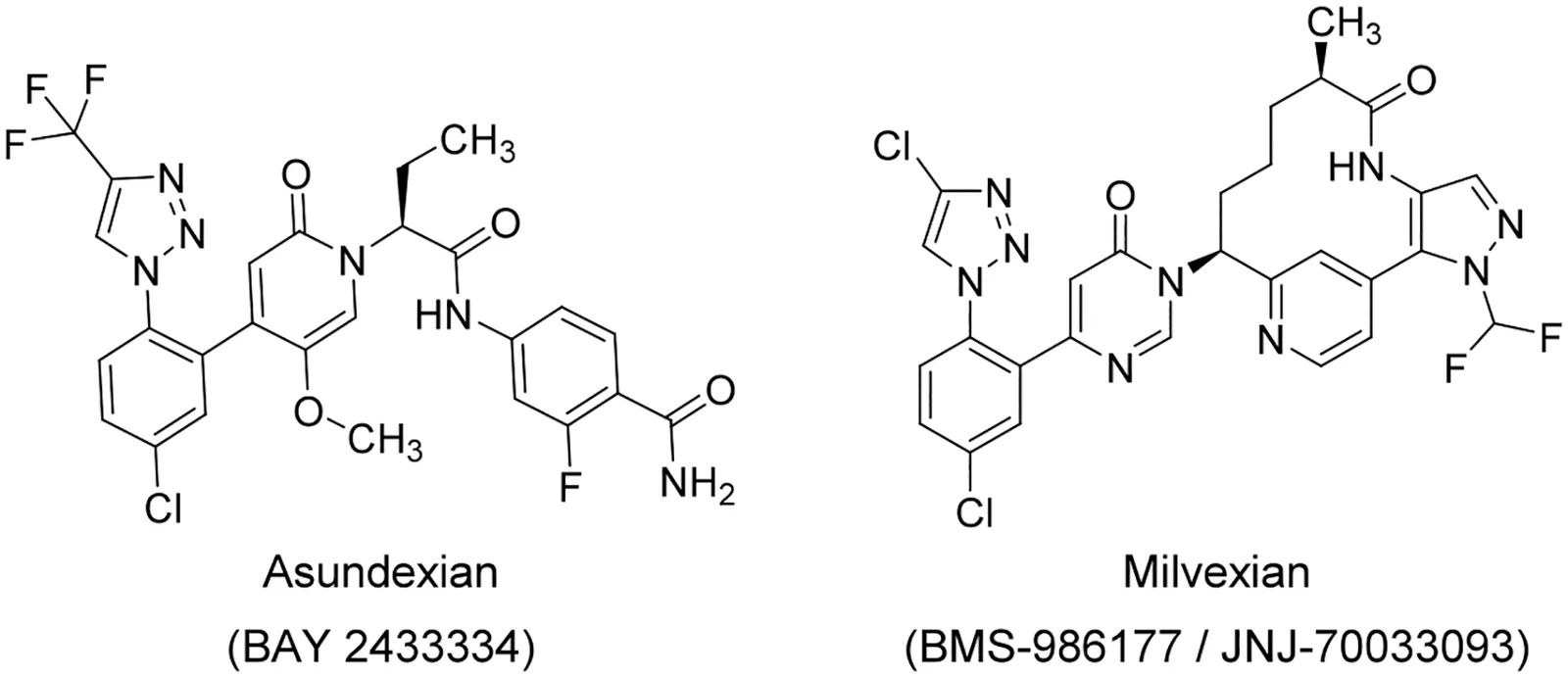
Chemical structures of oral small molecules of FXIa.

**Table 1.** Oral Small Molecule Inhibitor of FXIa^a^

Characteristics	Asundexian^41,45–49^ (BAY 2433334)	Milvexian^50–58,60^ (BMS-986177/JNJ-70033093)
***K***_***i***_ **(inhibitory constant)**	~0.26 nM	0.11 nM
**Selectivity**	>1,000-fold against other serine proteases	>1,000-fold against other serine proteases
**Oral bioavailability**	~94%	58%
**Elimination half-life**	~14–18 h	~8.3–13.8 h
**Apparent volume of distribution**	~70.5 L (apparent volume of distribution at steady state)	347 L (apparent volume of distribution, terminal phase)
**Apparent oral clearance**	2.56 L/h	18.6 L/h
	(CV 20.5%; ≈43 mL/min)	(CV 90.6%; ≈310 mL/min)
**Plasma protein binding**	~94%	~92%
**Metabolism**	CES1 (primary); CYP3A4 (minor)	CYP3A4 (primary)
**Excretion**	Fecal ~80%; urinary ~20% (6% unmodified)	Fecal; urinary <20% (7–18% unmodified)
**T**_**max**_	1–1.5 h (oral solution); 2–4 h (IR tablets)	2.5–4 h (fasted); 7–8 h (after meal, high doses)
**Dosing frequency**	Once daily (QD)	Twice daily (BID)
**Food effect**	Modest (AUC not clinically affected)	Pronounced at high doses
**P-glycoprotein substrate**	Yes	Yes
**Strong CYP3A4 and P-gp inhibitors**	AUC is increased by 103% (itraconazole); +76% (verapamil)	AUC is increased by 2.5-fold (itraconazole)
**Moderate CYP3A4 inhibitors**	No meaningful effect (fluconazole)	AUC is increased by 38% (diltiazem)
**CYP3A4 and P-gp inducers**	AUC is reduced by 44% (carbamazepine)	AUC is reduced to ~15% (rifampin)
**Renal impairment**	No dose adjustment required	AUC +41–54%; no adjustment expected; *t*_1/2_ prolonged to ~18 h
**Hepatic impairment**	No clinically relevant changes (Child-Pugh A/B)	Modest changes; no adjustment expected

a AUC, area under the concentration–time curve; BID, twice daily; CYP, cytochrome P450; IR, immediate release; P-gp, P-glycoprotein; QD, once daily; *T*_max_, time to maximum plasma concentration.

**Table 2.** Properties of Factor XI(a)-Directed Monoclonal Antibodies^a^

Characteristics	Abelacimab (MAA868)^69–72^	Osocimab (BAY1213790)^73–76^
**Form**	Fully human IgG	Fully human IgG
**Target/epitope**	FXI/FXIa catalytic domain	FXIa active-site adjacent region
**Route of administration**	IV and SC	IV and SC
**Dosing and Frequency**	150 mg IV loading and monthly SC; 90 or 150 mg monthly SC (AZALEA-TIMI 71); Monthly SC (after IV load)	0.3, 0.6, 1.2, 1.8 mg/kg IV single dose (FOXTROT); Monthly or peri-procedural IV/SC
**SC Bioavailability**	85%	75–85%
**Elimination half-life**	25–30 days (~600–720 h)	30–44 days (~720–1056 h)
**Volume of distribution**	Vdss 8.48 L (IV); 10.5 L (SC)	Vdss 5.50–10.3 L (IV); Vd(area)/F ~13.3–16.5 L (SC)
**Metabolism**	Proteolysis by the RES	Proteolysis by the RES
**Clearance**	ClB IV: 0.0102 L/h (0.17 mL/min); SC Cl/F: 0.0124 L/h (0.21 mL/min)	ClB IV: 6.41–13.5 mL/h (0.11–0.23 mL/min); SC Cl/F: 10.8–15.8 mL/h
**Renal/hepatic clearance**	None	None
**T**_**max**_	~1 h (IV); ~7–21 days (SC)	~1–3 h (IV); ~4–6 days (SC)
**CYP450/P-gp substrate**	No	No
**Effect on free FXI**	>99% reduction within 1 h of IV infusion; sustained reductions of 99% (150 mg) and 97% (90 mg) at 3 months with monthly SC dosing (AZALEA)	FXI activity reduction sustained for weeks after a single dose

a *Cl*B, total body (plasma) clearance after intravenous administration; Cl/F, apparent clearance after subcutaneous administration; RES, reticuloendothelial system; SC, subcutaneous; Vd(area)/F, apparent volume of distribution during the terminal phase after extravascular administration; Vdss, apparent volume of distribution at steady state. Others were mentioned in Table 1.

**Table 3.** Properties of Factor XI-Targeted ASOs^a^

Characteristics	IONIS-FXIRx^21,65,72,74,76^ (ISIS-416858; BAY2306001)	Fesomersen^72,74,76,82^ (IONIS-FXI-LRx; FXI-LICA; BAY2976217)
**Drug class**	second-generation 2′-MOE antisense oligonucleotide (unconjugated)	GalNAc3-conjugated 2′-MOE antisense oligonucleotide
**Route of administration**	Subcutaneous	Subcutaneous
**Elimination half-life**	~1–2 weeks (~168–336 h)	~10–20 days (~240–480 h)
**Plasma protein binding**	High (>90%)	High (>90%)
**Metabolism**	Endonuclease/exonuclease-mediated	Endonuclease/exonuclease-mediated
**T**_**max**_	~6 h	~1.5–3.5 h
**Dosing frequency**	200–300 mg subcutaneous weekly (FXI-ASO TKA trial)	40–120 mg subcutaneous monthly (RE-THINC trial)
**CYP450 substrate**	No	No
**P-glycoprotein substrate**	No	No
**Renal excretion**	Minimal (<1% recovered unchanged in urine)	~25% recovered in urine and ~72% in feces after subcutaneous radiolabeled dosing; predominantly hepatic uptake and catabolism
**Effect on circulating FXI**	Dose-dependent reduction of plasma FXI; maximal effect reached only after prolonged administration (weeks)	In RE-THINC (hemodialysis patients), median steady-state FXI reduction: 53.6% (40 mg), 71.3% (80 mg), and 86.0% (120 mg) vs 1.9% with placebo
**Onset/offset**	Slow onset (days–weeks); slow offset (half-life-driven)	Slow onset (days–weeks); slow offset (half-life-driven)
**Renal impairment**	No dose adjustment needed	No dose adjustment needed
**Hepatic impairment**	No reported studies	No reported studies

a GalNAc3, triantennary N-acetylgalactosamine; 2′-MOE, 2′-O-methoxyethyl. Others are mentioned above.

## ABBREVIATIONS

FXI: Factor XI
DALYs: disability-adjusted life years
ICH: intracranial hemorrhage
ESUS: embolic stroke of undetermined source
TF: tissue factor
FXII: factor XII
FX: factor X
VKAs: vitamin K antagonists
DOACs: direct oral anticoagulants
ASOs: antisense oligonucleotides
AUC: area under the concentration–time curve
BID: twice daily
CYP450: cytochrome P450
IR: immediate-release
P-gp: P-glycoprotein
QD: once daily
Tmax: time to maximum plasma concentration
CES1: carboxylesterase 1
RES: reticuloendothelial system
SC: subcutaneous
CMBs: cerebral microbleeds
hsCRP: high-sensitivity C-reactive protein
siRNA: small interfering RNA
